# Modelling the progression of pandemic influenza A (H1N1) in Vietnam and the opportunities for reassortment with other influenza viruses

**DOI:** 10.1186/1741-7015-7-43

**Published:** 2009-09-03

**Authors:** Maciej F Boni, Bui Huu Manh, Pham Quang Thai, Jeremy Farrar, Tran Tinh Hien, Nguyen Tran Hien, Nguyen Van Kinh, Peter Horby

**Affiliations:** 1 Oxford University Clinical Research Unit, Vietnam; 2 MRC Centre for Genomics and Global Health, University of Oxford, Oxford, UK; 3 National Institute of Hygiene and Epidemiology, Hanoi, Vietnam; 4 Centre for Tropical Medicine, Nuffield Department of Clinical Medicine, University of Oxford, Oxford, UK; 5 Hospital for Tropical Diseases, Ho Chi Minh City, Vietnam; 6 National Institute for Infectious and Tropical Diseases, Hanoi, Vietnam; 7 South East Asia Infectious Disease Clinical Research Network, Vietnam

## Abstract

**Background:**

A novel variant of influenza A (H1N1) is causing a pandemic and, although the illness is usually mild, there are concerns that its virulence could change through reassortment with other influenza viruses. This is of greater concern in parts of Southeast Asia, where the population density is high, influenza is less seasonal, human-animal contact is common and avian influenza is still endemic.

**Methods:**

We developed an age- and spatially-structured mathematical model in order to estimate the potential impact of pandemic H1N1 in Vietnam and the opportunities for reassortment with animal influenza viruses. The model tracks human infection among domestic animal owners and non-owners and also estimates the numbers of animals may be exposed to infected humans.

**Results:**

In the absence of effective interventions, the model predicts that the introduction of pandemic H1N1 will result in an epidemic that spreads to half of Vietnam's provinces within 57 days (interquartile range (IQR): 45-86.5) and peaks 81 days after introduction (IQR: 62.5-121 days). For the current published range of the 2009 H1N1 influenza's basic reproductive number (1.2-3.1), we estimate a median of 410,000 cases among swine owners (IQR: 220,000-670,000) with 460,000 exposed swine (IQR: 260,000-740,000), 350,000 cases among chicken owners (IQR: 170,000-630,000) with 3.7 million exposed chickens (IQR: 1.9 M-6.4 M), and 51,000 cases among duck owners (IQR: 24,000 - 96,000), with 1.2 million exposed ducks (IQR: 0.6 M-2.1 M). The median number of overall human infections in Vietnam for this range of the basic reproductive number is 6.4 million (IQR: 4.4 M-8.0 M).

**Conclusion:**

It is likely that, in the absence of effective interventions, the introduction of a novel H1N1 into a densely populated country such as Vietnam will result in a widespread epidemic. A large epidemic in a country with intense human-animal interaction and continued co-circulation of other seasonal and avian viruses would provide substantial opportunities for H1N1 to acquire new genes.

## Background

In early 2009 a novel influenza A (H1N1) variant emerged which spread globally causing the first influenza pandemic in over 40 years. The dynamics and impact of this pandemic are difficult to predict, especially since the world has changed significantly in 40 years - the global population has almost doubled, more people live in cities, people travel more frequently and over longer distances. These facts will undoubtedly influence the global pattern of this pandemic, just as geographical heterogeneities will result in different local patterns [1]. More than 60% of the world's population live in low-income and lower-middle income countries, and yet, at the time of writing only, about 10% of confirmed cases have occurred in these areas [2,3]. In densely populated low-income countries, where public health systems, health care services and drug availability are all stretched, influenza H1N1 is likely to be almost impossible to contain resulting in a greater number of cases occurring in more vulnerable populations resulting in a less benign epidemic.

Even more worrying, almost 60% of the world's human population and over 50% of the world's poultry population live in Asia, where highly pathogenic avian influenza (HPAI) maintains a foothold and seasonal influenza transmission is complex [4]. Previous pandemics have demonstrated the potential consequences of reassortment between human and animal influenza viruses. It is possible, therefore, that the new H1N1 - itself a reassortant of swine viruses that had previously reassorted with human and avian influenza - may follow a similar pattern [5,6]. The new H1N1 variant has already shown that it can be transmitted from humans to pigs, and we know that the H5N1 subtype is capable of infecting humans and of successfully reassorting with human seasonal influenza viruses under *in vitro *and *in vivo *experimental conditions [7,8]. As the population is increasing and standards of living are improving there has been an increase in livestock production and thus there is probably more contact between animals and humans than before. These contacts offer opportunities for reassortment, and, if a novel virus with the transmissibility of H1N1 and even a fraction of the virulence of H5N1 were to emerge, the consequences would be devastating.

In order to explore the potential impact of influenza A (H1N1) on a densely populated low-income country, we developed a mathematical model showing how an influenza A (H1N1) epidemic might progress in Vietnam. We used this model to estimate the frequency of contact between H1N1 infected humans and domestic animals in an attempt to quantify the opportunities for reassortment between H1N1 and animal influenza viruses.

## Methods

### Mathematical model

We developed an age-structured gravity model - where migration rates among sub-populations are balanced such that there are no changes in the sizes of the sub-populations - based on traditional susceptible exposed infectious recovered (SEIR) equations with stochastic migration and hospitalization processes [9]. The model has geographical resolution to the province level in Vietnam (64 provinces in 2007) and tracks infection and mixing in seven age groups. The incubation period was set at 1.4 days and the infectious stage was separated into four stages to mimic an infectious period that is Γ-distributed with a coefficient of variation equal to 0.5. Mixing and infection among hosts (humans) in the model occurred at the province level and depended on the contact rates among the seven age groups, age-specific susceptibilities, province-specific age distribution and population density. The basic reproductive number, denoted by *R*_0_, is calculated via a next generation matrix assuming at most one cross-province migration event during a single infection [10]. The *R*_0 _value described in the text and figures is for Ho Chi Minh City and assumes that there is no migration from the city (see supplementary materials, additional file 1, for detail on the different *R*_0 _values that can be computed for this model). The results are presented for a single case introduced in Ho Chi Minh City, as this is where the first case was confirmed on 31 May 2009. Infection among animal populations is not modelled. Model equations and details of computing the basic reproduction ratio are presented in the supplementary materials (Additional file 1).

### Data sources

We used seven age groups: 0-5 years, 6-15, 16-25, 26-34, 35-49, 50-64 and 65+. Provincial level data on resident population by age class, number of public and private hospital beds, number of households, and number of households raising pigs, chickens, and ducks were derived from the General Statistics Office of Vietnam. The age-class specific daily probability of migration between provinces was derived from a 2007 community survey conducted in northern Vietnam [unpublished data, P Horby]. This gave a mean estimate of 1.35% of the population moving between the provinces each day. This was used as the lower end of the modelled range, as it is known that populations closer to urban areas will have much higher rates of movement. The number of major and minor roads crossing provincial borders was determined from 1:250,000 road maps and were used to obtain a relative measure of interprovincial traffic. Internal migration by air travel was estimated using publicly available flight data from all airlines operating domestically in Vietnam. The known daily travel by air and the unknown daily travel by road were combined to form a scalable migration network between the provinces of Vietnam where between 1.35% and 5.00% of the population moved between provinces on a daily basis.

### Transmission and natural history parameters

Age-dependent mixing was included in the model by creating a contact matrix for seven age groups, using data from a survey of social contact patterns conducted in 2007 among 865 members of a community in one semi-rural district of northern Vietnam. Since both epidemiological and serological data are suggestive of age-dependent susceptibility to H1N1 infection, an age-dependent susceptibility term was also included [11,12]. This was derived using data on the age distribution of cases in the USA and data on age-dependent contact frequency from a European study [13]. We assumed no effect of season on the transmission of infection or on contact patterns, as influenza seasonality in Vietnam is not well understood, even in the northern and more temperate part of the country (unpublished data, PQ Thai).

Since reliable data on the natural history of infection with H1N1 were not available at the time of writing, we applied parameters previously estimated for seasonal influenza. We applied an incubation period with a mean of 1.4 days [14]. The mean of the Γ-distributed infectious period was varied between 3.8 days and 5.5 days [15]. The age-class specific relative probability of hospitalization was derived from data of the proportion of H1N1 cases hospitalized in Mexico and the USA. The overall hospitalization rate was varied between 0.5% and 1.5% of all cases, since reported rates of 5%-6% are likely to be biased by over-ascertainment of severe cases compared to mild cases. Hospitalization time was set at 5 days [16].

### Sensitivity analysis

Sensitivity was tested by varying the basic reproduction ratio (1.2 - 3.1), the duration of infection (3.8 - 5.5 days), the individual probability of cross-province migration (1.35% - 5.00% daily probability), the relative amount of traffic on large roads compared to small roads (one to two times), and the overall expected hospitalization in the population (0.5% - 1.5%). One thousand parameter combinations were sampled using Latin hypercube sampling, and sensitivity results are reported for these parameter samples [17]. The key parameter for this sensitivity analysis is *R*_0_, the basic reproductive number. For influenza this is traditionally estimated between 1 and 3 [18-20] and the ranges reported so far for novel H1N1 have been 1.2, 1.4 to 1.6, 2.0 to 2.6, and <2.2 to 3.1 [11,21,22]. For the upper band of our tested range, we used the highest estimate (*R*_0 _= 3.1) as opposed to the highest of the upper band 95% confidence interval (*R*_0 _= 3.5).

Full details of data sources, parameter estimation and model specification are available in the supplementary materials (Additional file 1).

## Results

### Epidemic curve and geographic spread

Introducing a single infected case in Ho Chi Minh City, and simulating the epidemic for one year (over 1000 randomly sampled parameter sets), resulted in a median 6.4 million infections (IQR: 4.4 million - 8.0 million). In the absence of any intervention, the epidemic would reach half of Vietnam's provinces in 57 days (IQR: 45-86.5), and would peak after 81 days (IQR: 62.5-121). Seventy-seven percent of all cases and 67% of all hospitalizations occur in the 6-34 year age group. Table 1 shows the range of outputs for the model simulations.

**Table 1:** Median, quartile and minimum - maximum values for selected outputs of one year of model simulation.

**Model output**	**Minimum**	**Lower quartile**	**Median**	**Upper quartile**	**Maximum**
Time to reach 20-case point (days)	9.0	12.0	14.0	19.0	39.0

Time to reach 100-case point (days)	13.0	18.0	22.0	32.0	71.0

Time for 32 provinces to be affected (days)	34.0	45.0	57.0	86.5	314.0

Time for 48 provinces to be affected (days)	41.0	55.0	71.5	112.0	> 1 year

Epidemic peak point (days)	45.0	62.5	80.8	121.0	not reached

Final epidemic size (number of cases)	103,885	4,432,247	6,377,555	8,021,328	9,796,738

Cumulative number hospitalized	594	38,832	58,165	74,935	104.976

Average number of new cases per day over 2-week peak period	1916	88,453	174,804	245,209	326,260

Average number of new hospitalizations per day over 2-week peak period	9	779	1,564	2,238	3508

Cumulative number of cases in swine owners	1940	224,208	410,276	671,703	1,159,291

Cumulative number of cases in chicken owners	630	172,731	351,243	632,316	1,174,682

Cumulative number of cases in duck owners	160	23,732	51,131	95,790	182,520

Number of days Hanoi hospitals running at > 150% bed capacity	0	0	0	14	20

Number of days HCMC hospitals running at > 150% bed capacity	0	0	0	0	11

Time from 100-case point in HCMC to 100-case point in Hanoi	12	23	29	43	156

Number of rural cases	16,105	963,791	1,540,008	2,184,359	3,220,171

Number of urban cases	87,780	3,440,732	4,844,258	5,831,431	6,668,559

Number of exposed pigs	3,053	259,328	462,633	737,443	1,239,324

Number of exposed chickens	4,612	1,890,957	3,745,045	6,417,106	11,419,922

Number of exposed ducks	2,319	580,132	1,176,129	2,072,495	3,708,187

Number of infections by age group					

0 to 5 years	7,611	366,741	543,858	685,656	832,651

6 to 15 years	35,370	1,211,114	1,617,223	2,031,725	2,609,789

16 to 25 years	30,075	1,312,009	1,837,835	2,212,661	2,599,191

26 to 34 years	22,277	998,079	1,450,723	1,784,407	2,096,515

35 to 49 years	6,067	363,076	627,659	870,774	1,134,289

50 to 64 years	1,920	130,147	232,582	329,305	435,306

65 years and over	565	38,492	71,658	106,032	147,498

Hospitalizations by age group					

0 to 5 years	71	4,493	6,766	8,624	12,144

6 to 15 years	113	5,453	7,467	9,481	13,831

16 to 25 years	119	8,128	11,393	14,091	18,991

26 to 34 years	198	13,580	19,932	24,989	33,959

35 to 49 years	81	5,540	9,424	13,345	19,910

50 to 64 years	10	1,284	2,279	3,310	5,262

65 years and over	2	393	721	1,079	1,792

**Figure 1:**
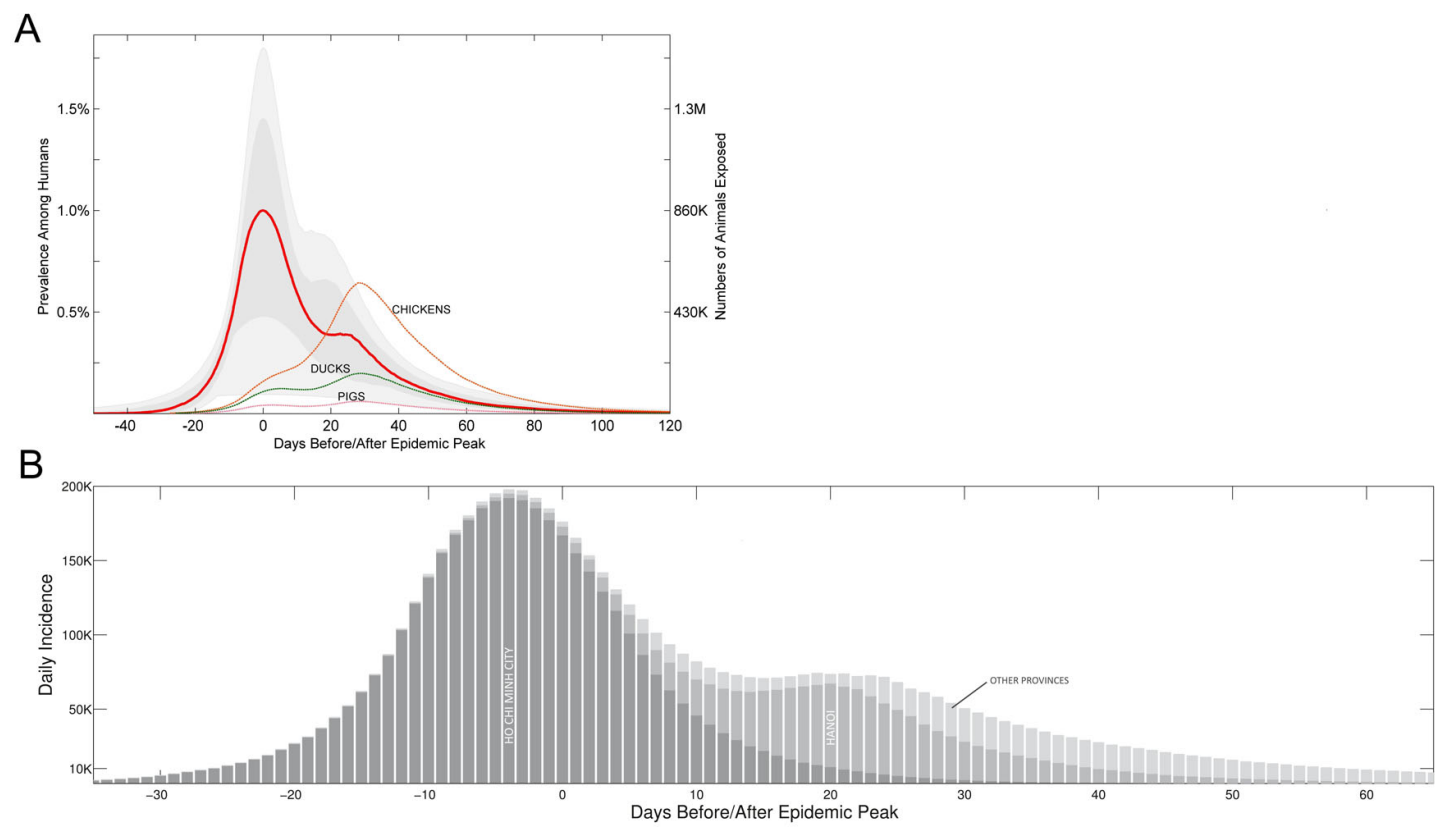
**(A) The range of possible epidemics in Vietnam**. The graph summarizes 500 simulated epidemics and resets their peaks to day zero so they can be compared on the same time axis. The red line shows the median number of infected persons. The medium gray region shows the interquartile range. The light gray region shows the 95% confidence interval based on the parameter ranges chosen via Latin hypercube sampling. The confidence band width is primarily determined by *R*_0_. The three dotted lines show the median number of exposed animals during the epidemic. **(B) Median number of new cases by day, with day zero corresponding to the epidemic peak**. Stacked bar graph has dark gray bars for Ho Chi Minh City, medium gray bars for Hanoi and light gray bars for the remaining 62 provinces.

**Figure 2:**
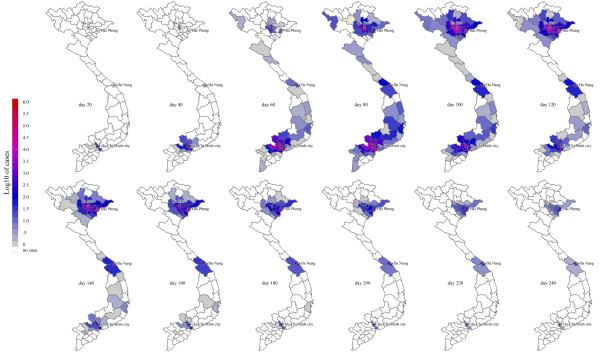
**Geographic spread of swine-origin influenza A (H1N1) in Vietnam**. Case numbers in each province are medians from 1000 model simulations. See additional file 2 for corresponding animation.

**Figure 3:**
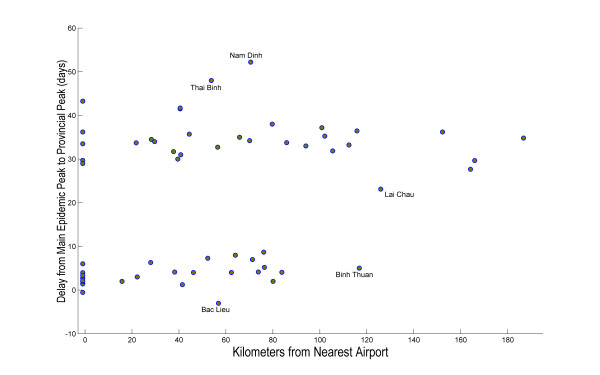
**Timing of provincial epidemic peaks based on the distance from the nearest airport to the capital city**. The model does not take sub-provincial population structure into account, and the epidemic's progression is determined primarily by south-to-north movement rather than distance to the airport network. Binh Thuan has an early peak because it lies in a densely populated part of southern Vietnam. The Lai Chau peak, as estimated by our model, probably occurs too early. Lai Chau is remote and sparsely populated, but its adjacency to the Dien Bien Phu airport causes the model to predict an early epidemic peak.

The epidemic was dominated by the peaks in Hanoi and Ho Chi Minh City (Figure 1), Vietnam's most densely populated metropolitan areas. Both of these provinces are at least twice as densely populated as any other province in Vietnam. The interval between the 100-case point in Ho Chi Minh City and 100-case point in Hanoi is estimated to be about 29 days (IQR: 23-43), but might be doubled or tripled if a sustained social distancing campaign were able to reduce all contacts by 50%. After the Hanoi wave passes, the epidemic is expected to tail off slowly as the disease spreads to less densely populated rural areas. Figure 2 shows the geographic progression of the median epidemic in Vietnam; Figure 3 shows the median epidemic peak times for all the provinces, indicating an approximate 1-month delay between peaks in the southern provinces and peaks in the northern provinces.

The epidemic in Vietnam is predicted to cause 58,000 hospitalizations (IQR: 39,000-75,000). The health care system would be severely stretched but is unlikely to be overwhelmed, except in the case of a high-*R*_0 _epidemic or increased virulence. Vietnam currently has a stockpile of approximately 1.1 million oseltamivir treatment courses (10 75 mg tablets) and sufficient powder to formulate another 900,000 treatment courses. This should be adequate for treatment of severe cases but for not mild cases or for prophylaxis of contacts during a widespread epidemic.

### Contacts between infected humans and domestic animals

**Figure 4:**
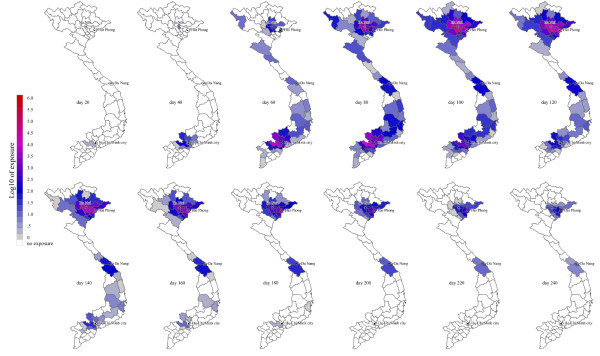
**Geographic timeline of chicken exposure during an influenza epidemic in Vietnam**. The numbers of chicken exposures are medians from 1000 models simulations. Duck and pig exposures were highly correlated with chicken exposures, geographically and temporally. Note that because of rounding and fractional cases, some sparsely-populated provinces may have a median of 0.2 human cases (rounded down to zero) and 0.8 chicken exposures (rounded up to 1). See additional file 3 for corresponding animation.

Because of the slow dispersion of the epidemic into rural areas, the peak exposure of domestic pigs, ducks and chickens to infected humans occurs during the later phases of the epidemic. Figure 1A shows the estimated number of exposures of domestic animals to infected humans; the highest exposure will be among domestic chickens and the exposure of all domestic animals will peak roughly 1 month after the peak in Ho Chi Minh City and shortly after the epidemic peak in Hanoi. Note that the tail phase of the epidemic wanes slowly and that a significant number of chickens, ducks and pigs remain exposed for up to 2 months after the human epidemic has peaked in Hanoi (Figure 4).

In total, the epidemic simulations estimate a median 410,000 cases among swine owners (IQR: 220,000 - 670,000) with 460,000 exposed swine (IQR: 260,000-740,000), a median 350,000 cases among chicken owners (IQR: 170,000-630,000) with 3.7 million exposed chickens (IQR: 1.9 M-6.4 M), and a median 51,000 cases among duck owners (IQR: 24,000 - 96,000), with 1.2 million exposed ducks (IQR: 0.6 M-2.1 M).

### Effect of public health interventions

By restricting contacts in the 6-15 age group, school closures were modelled but showed little effect on the progression of the epidemic. Even a comprehensive strategy of restricting all contacts within this age group would only delay the epidemic peak by a few days and result in no fewer cases. Any realistic restriction of flights between Ho Chi Minh City and Hanoi (< 2 weeks) had little or no effect on geographic spread or the total number of cases. Monitoring incoming international flights and multiple introductions was not modelled.

### Sensitivity analysis

**Figure 5:**
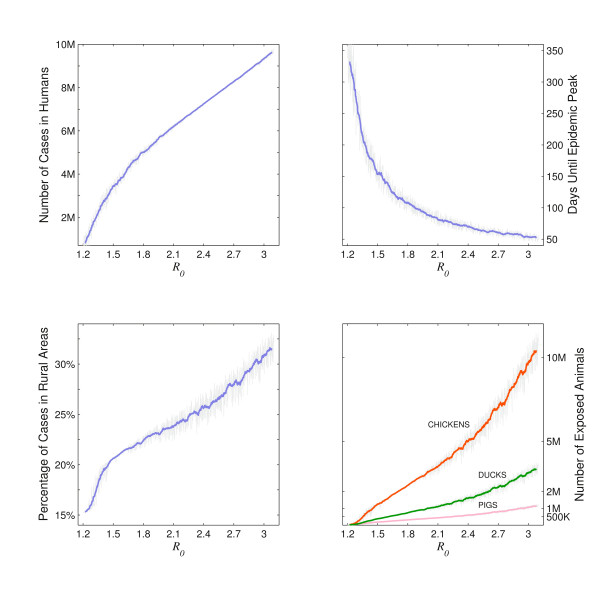
**Result sensitivity relative to the *R*_0_-value as it would have been measured in Ho Chi Minh City**. Light gray lines show the variation in a particular epidemiological indicator as a function of *R*_0_. The other coloured lines are moving averages over nearby *R*_0_-values. The top two panels show the size of the epidemic and the time taken for it to peak, which always have a predictable relationship to *R*_0_. The bottom two panels show how animal exposure increases and how the epidemic becomes more rural as *R*_0 _increases. Note that with higher *R*_0_, not only does the risk to domestic animal owners increase but the relative risk of an owner to a non-owner also increases (not shown).

Like all epidemic models, the highest sensitivity is to *R*_0_. All severity indices of the epidemic - total number of cases, peak incidence and total number of hospitalizations - rise steadily with the *R*_0 _value, or, in general, with the transmissibility of the virus (top panels, Figure 5). The most important feature of the model is that with increasing *R*_0 _the epidemic becomes more rural. An increase in the predicted transmissibility of novel H1N1 in Vietnam results not only in more infections, but in a higher proportion of infection among rural populations and among those raising pigs, ducks and chickens domestically (bottom panels, Figure 5). The model is not very sensitive to the other parameters tested: the duration of the infection, the amount of migration between the provinces, the hospitalization rate or the relative amount of traffic on large roads versus small roads.

## Discussion

The first cases of H1N1 were detected in Vietnam on 31 May 2009 and by mid-July there were more than 100 confirmed cases with probable community transmission that was most likely the initial budding of the coming nationwide epidemic. We have used a mathematical model to explore how the epidemic might progress in the absence of interventions and have estimated the number of pigs, ducks and chickens that might be exposed to infected humans during the epidemic. Employing mathematical modelling for such a forecasting exercise comes with many caveats. Of these, the most important are that real individuals are heterogeneous in behaviour and transmission, that human behaviour can change as a result of the severity of the epidemic and that the spatial dimensions of transmission have many nested levels that may or may not alter the progression of the epidemic on a larger scale [23,24]. We used a 'patch model' with coarse province-level spatial resolution for simplicity of model development and rapid computation; the model results should, therefore, be viewed as rough estimates of the epidemic's impact in Vietnam on a year-long time scale.

The most important caveat in our analysis is that the true basic reproductive number is not known; we used a conservative estimate, between 1.2 and 3.1, based on early measurements taken in Mexico, USA and Japan, and we stress that the *R*_0 _for Vietnam may be higher than these estimates. For an *R*_0 _value of 4.0, our model predicted a total of 13.3 million cases among humans; for an *R*_0 _value of 5.0, 16.6 million cases were predicted. Unfortunately, the uncertainty in Vietnam's *R*_0 _will not be resolved until we analyse the progression of cases from the first wave of this pandemic.

Although the model predicts substantially more cases than have so far been reported from other H1N1 affected countries, the clinical illness is predominantly mild and, therefore, reported H1N1 cases to date reflect only a small proportion of the total number of cases. Our modelled epidemic affects a median of 7.4% of the population (IQR: 5.2-9.3%). This rate is below the figures for previous pandemics and might be considered too low given the high transmissibility of this virus in some settings and the expectation that most of the population would have no immunity [12]. Due to the low probability of hospitalization, it is unlikely that the health sector as a whole will be over-whelmed in the scenario outlined in this model. However, there is considerable variation in reported hospitalization rates for H1N1 and the estimate of 1% that we have used is considerably lower than the maximum of 6% [25]. As elsewhere, the number of intensive care beds is limited in Vietnam and occupancy is routinely at maximum; therefore intensive care capacity is likely to be easily overwhelmed. Also, although Vietnam has impressive health indicators for its economic status - the population may have vulnerabilities, such as under-nutrition in children, which might result in a greater number of severe cases than observed elsewhere.

Containment does appear to have been temporarily successful in some countries (Mexico and Japan) but not in others (Australia and the USA). The reasons for these differences are undoubtedly complex, but successful case detection, isolation and treatment, quarantine and chemoprophylaxis of contacts, and social distancing measures, may all have an effect on the results. In our model, school closures did not make a substantial difference to the epidemic progression, although substantial decreases in contact frequency across all age groups would delay the time course of the epidemic. School outbreaks have been a major feature in the early stages of this pandemic, and it is possible that our model underestimates the role of the range of contacts and susceptibility of school-age children on the epidemic dynamics. School closures did seem to be effective in Kobe, Japan, during 11-24 May 2009, but this may have reflected the low number of overall infections in Japan at that time (between four and 345 confirmed cases) [26]. In the UK, a plateau in consultation rates appears to have coincided with the closure of schools for the annual summer holidays [27]. Previous work suggests that school closure can modify peak attack rates and may result in a modest reduction of the final number of cases, but empiric data is still required on the effectiveness of school closure on reducing the number of transmissions [28-31]. Climate and other seasonally variable factors may also have acted to limit transmission in temperate regions [32,33]. Seasonal factors are likely to have less influence in tropical regions where the seasonality of influenza transmission is much less marked [4].

In the absence of effective interventions, we predict a large amount of contact between infected humans and animals that might harbour other influenza viruses, including HPAI. In fact, we believe our model probably underestimates the amount of contact between infected human and animals for three reasons. First, we divided the total number of human cases by the number of people per household in order to derive an estimate of the number of households with an infectious case. We did this to avoid over counting animals that were exposed to multiple infected individuals in the same household, but this is a very conservative correction. Second, domestic animal production is concentrated close to urban centres, where population densities are higher than average. Third, we did not model contacts which occurring in live poultry markets or commercial farms.

The danger of human-animal contact lies in the opportunity for reassortment among different influenza subtypes. It is well known that influenza reassorts in humans [34], that pigs play an important role in reassortment of human/avian/swine influenza viruses [35-37] and that the history of avian influenza viruses includes multiple reassortment events [38,39]. However, very little is known about the potential of human influenza viruses to jump to animals, since most studies to date have focused on animal influenza activity and the risk it poses to humans [40-42]. Pandemic H1N1 has already been detected in swine and, since poultry and swine populations in Asia may harbour many different subtypes of influenza (at least H4, H5, H6, H7, H9, H11, H12), the generation of a new subtype through a reassortment event is a real possibility [43,44] [personal communication, Ken Inui].

Although these opportunities for genetic reassortment are not unique, the current influenza landscape contains worrying features. Widespread epidemics of novel H1N1 are likely in tropical countries where HPAI is endemic and seasonal influenza transmission is complex and sustained, without the seasonal bottlenecks that characterize transmission in temperate regions [4,33]. The overall diversity of influenza viruses in southeastern Asia ensures that an epidemic of the novel H1N1 will create many opportunities for co-infection with other subtypes circulating in the region. Genetic and antigenic data suggest that Asia is a key source of influenza viruses that cause seasonal outbreaks in the northern and southern hemispheres [45]. This region, therefore, possesses the conditions necessary for the genesis and dissemination of new influenza variants [33,45]. Finally, the introduction of H1N1 into southeastern Asia creates an optimal evolutionarily environment for the virus, where re-assortment is neither too frequent nor too rare [46]. This means the virus receives the benefits of limited reassortment (a genetic novelty) but not the penalty of high levels of reassortment (the breaking apart of beneficial gene combinations).

Our model provides a rough picture of what might happen in Vietnam, but it includes many assumptions, uncertainties and un-modelled heterogeneities which require that the results be interpreted with caution. Although changes in human demography and migration over the past 40 years may make a pandemic more difficult to control, the same period has seen massive advances in technology and communication that allow us to monitor and predict this pandemic as never before. Mathematical models are one tool, but a criticism of these models is that the predictions are not subsequently tested against real outbreak data [47]. Our model development has coincided with the arrival of H1N1 in Vietnam and we are planning to track the progression of the outbreak in Vietnam in an attempt at real-time model validation and diagnostics.

## Abbreviations

IQR: interquartile range; HPAI: highly pathogenic avian influenza; SEIR: susceptible exposed infections recovered.

## Competing interests

The authors declare that they have no competing interests.

## Authors' contributions

PH and MFB conceived the study and designed the model structure. PH, BHM, PQT and MFB collated the data and model parameters. MFB wrote the model code and ran the model and sensitivity analysis. BHM prepared all maps and video sequences. TTH, NTH, NVK and JF provided data and advised on the model design. PH and MFB wrote the first draft of the paper. All the authors reviewed and edited drafts of the manuscript and approved the final version. PH, BHM, MFB contributed equally.

## Pre-publication history

The pre-publication history for this paper can be accessed here:



## Supplementary Material

Additional file 1 **Supplementary materials**. Describes details of model construction, data sources, parameter estimation, *R*_0 _calculation, and sensitivity analysis. Click here for file

Additional file 2 **Geographic spread of swine-origin influenza A (H1N1) in Vietnam**. Animated GIF file that shows the full day-by-day epidemic shown in Figure 2. Click here for file

Additional file 3 **Geographic timeline of chicken exposure during an influenza epidemic in Vietnam**. Animated GIF file that shows the full day-by-day exposure of chickens to human influenza infections shown in Figure 4. Click here for file
