# Supplementary material for: Modelling the progression of pandemic influenza A (H1N1) in Vietnam and the opportunities for reassortment with other influenza viruses
Source: BMC Med. 2009 Sep 3;7:43. doi: 10.1186/1741-7015-7-43 (PMC2753341; doi:10.1186/1741-7015-7-43)
Supplement: Additional file 1 — Supplementary materials. Describes details of model construction, data sources, parameter estimation, R0 calculation, and sensitivity analysis. [file 1741-7015-7-43-S1.pdf]

Supplementary material

Spatial structure

The boundaries of the 64 Provinces of Vietnam in 2009 were obtained in GIS format from the Global Mapping Project [http://www.iscgm.org/cgi-bin/fswiki/wiki.cgi]. All disease dynamics and relevant population characteristics were aggregated at the province level.

Age structure

Seven age classes were used: 0-5 years, 6-15, 16-25, 26-34, 35-49, 50-64, 65+. These age classes were chosen since they correspond to the age classes used in a community survey of contact and travel frequency.

Population

The estimated resident population, rural and urban, in 2007 by Province was obtained from the Government Statistics Office of Vietnam (GSO). The 2007 population estimates are compiled from statistical returns to GSO from Provincial and District Statistical Offices. The estimated population by seven age classes was derived by applying the age structure from the 1999 population census (originally 18 age classes) to the 2007 figures. Province specific population density was estimated using 2007 population data and provincial land area calculated in GIS after excluding forested areas (as classified by 2005 vegetation map from Ministry of Agriculture and Rural Development).

Age dependent mixing

Age class specific contact frequency was derived from a survey of social contact patterns conducted in 2007 in 865 members of a community in one semi-rural district of north Vietnam. A contact was defined as:

Either

- a two-way conversation with three or more words in the physical presence of another person

Or

- physical skin-to-skin contact (for example a handshake, hug, kiss or contact sports).

Participants recorded every contact made during one day, the age of the contact, and the duration and location of each contact. If a person was contacted more than once in a day, the contact was recorded only once but the total time spent with that contact over the entire day was recorded. A contact intensity matrix by seven age classes was constructed by adjusting the daily contact frequency data by the size of each age class. The matrix was corrected for reciprocity, i.e. where the contact frequency between age classes was not symmetrical the mean of the two values was used for both. The data were normalized so that the all age-class specific rates were relative to the maximum rate of 1.

The contact rate matrix is

AGE CLASS	0 TO 5	6 TO 15	16 TO 25	26 TO 34	35 TO 49	50 TO 64	65 AND OLDER
0 TO 5	.5797	.1726	.2000	.4804	.1873	.1760	.0489
6 TO 15	.1726	.8722	.1986	.2331	.2495	.1193	.1427
16 TO 25	.2000	.1986	.6889	.2782	.3162	.3344	.1630
26 TO 34	.4804	.2331	.2782	.6777	.3713	.4990	.3141
35 TO 49	.1873	.2495	.3162	.3713	.4836	.4593	.3504
50 TO 64	.1760	.1193	.3344	.4990	.4593	1.000	.5606
65 AND OLDER	.0489	.1427	.1630	.3141	.3504	.5606	.9916

and it represents relative contact rates corrected for population size, meaning these are the relative contact rates one would observe if each of the age classes had the same number of individuals. The true contact patterns are different for each province since the age distribution in each province is somewhat different. This pattern is similar to one calculated from census data from Portland, Oregon (USA), in that the main areas of contact intensity are (1) within age-groups and (2) peaking in children and older adults [1].

Note that these contact patterns differ from the European data presented in Mossong et al, the main difference being the high intensity of contacts in the 50+ age groups in

Vietnam [2]. The next-generation matrix computed for these data is different for each province in Vietnam (since age structure varies by province), and it is generally different than the Dutch next-generation matrix presented in Wallinga et al [3]. According to these next-generation matrices, infection patterns in Vietnam would primarily be driven by the 6-15 age group, while infection in the Netherlands would be evenly driven by 6- to 39-year-olds.

Internal migration by land

Age class specific frequency of travel outside of the Province of residence was estimated from a questionnaire based survey of travel patterns conducted in 2007 in 865 members of a community in one semi-rural district of north Vietnam.

A 64 x 64 adjacency matrix was compiled for all 64 Provinces in Vietnam that recorded which Provinces share a common border. For each pair of adjacent Provinces, the number of major and minor roads crossing the common border was determined from 1:250.000 road maps. Key national highways were classified as major roads and other national roads, provincial roads, and bridges were classified as minor roads. Major roads were given a value of 1 and minor road value was varied between 0.5 and 1.0 in our sensitivity analysis; this was deemed a reasonable range of variation as we did not have any other measure of the amount of traffic on large roads relative to small roads. These values were entered into a 64 x 64 connectivity matrix. A special direct connection was created between Hanoi and Hai Phong (the major port in north Vietnam) in the model, since a large amount of road traffic travels between these two Provinces along one major road but the two Provinces share no adjacent border, so connectivity was thought to be under-estimated in the model.

Province and age-class specific frequency of travel overland outside of the Province of residence was estimated by adjusting the frequency of travel survey data by the relative connectivity of each Province.

Internal migration by waterways and railways was ignored in the model since GSO data on the volume of traffic by type of transport indicated that waterway and railway travel together contributed less than 10% of all passenger volume in 2007.

Internal migration by air

Internal migration by air travel was estimated using publicly available data on the frequency of flights and aircraft type between domestic airports in Vietnam. It was assumed that all flights were full since data on the number of passengers was not available. These data are publicly available for Vietnam Airlines (http://www.vietnamair.com.vn/wps/portal/vn/site/flight_info/time_table), JetStar (http://www.jetstar.com/vn/vi/cheap-flights/standard.aspx), and Indochina Airlines (http://www.indochinaairlines.vn/FliSchedule.aspx).

Disease natural history

Data on the natural history of novel H1N1 were derived from published surveillance, epidemiological and clinical data and from a model describing the pandemic potential of H1N1 [4]. See table S1 for parameter values used:

Table S1

Parameter	Source	Value
Incubation period	[5]	Mean 1.4<br>(exponential distribution mimicked via standard linear term in differential equations)
Infectious period	[6]	Mean varied between 3.8 days and 5.5 days<br>(gamma distribution mimicked by four infectious compartments in SEIR model)
Basic reproduction ratio (number of secondary cases created by a single infectious case introduced into a fully susceptible population)	1.4-1.6 (genetic analysis 1.2)<br>[4]<br>Less than 2.2-3.1<br>[7]<br>Japan 2.3 (2.0-2.6)<br>[8]<br>School outbreak in the U.S. 2.69 (2.20-3.2)<br>ProMED-mail Influenza A (H1N1) - worldwide (51): 20090529.1999	Range 1.2 – 3.1

The overall hospitalization rate was set at 1% and the distribution of these hospitalizations across the age-classes was derived from data of the proportion of H1N1 cases hospitalized in Mexico and the U.S by age [9]. In the model the overall hospitalization rate was varied between 0.5% and 1.5% of all cases, since reported rates of 5-6% are likely to be biased by over-ascertainment of severe cases compared to mild cases. (Table S2).

Table S2

Age class	Probability of a case being hospitalized
Age 0-5	0.013
Age 6-15	0.005
Age 16-25	0.006
Age 26-34	0.014
Age 35-49	0.016
Age 50-64	0.010
Age 65+	0.011

Age dependent susceptibility to infection

Fraser et al found that the model that best fit the available data included both an age-dependent contact intensity parameter and age dependent susceptibility to infection [4]. Studies of age specific serological reactivity against the novel H1N1 virus are consistent with an age-dependent susceptibility to infection or disease [10]. Since age-dependent contact intensity is represented in our model by the contact matrix described on page 2 of this supplement, we sought to estimate the contribution of age-dependent susceptibility. To do this we used data on the age distribution of cases in the US and data on age dependent contact frequency from a European study [2, 9]. The relative frequency of contact by age class was factored out of the relative distribution of H1N1 cases by age in the US in order to derive an estimate of susceptibility by age class, independent of contact behaviour (Table S3).

Table S3

Age class	Relative susceptibility to infection
Age 0-5	0.77
Age 6-15	1.00
Age 16-25	0.82
Age 26-34	0.59
Age 35-49	0.19
Age 50-64	0.17
Age 65+	0.10

Seasonality

We assumed no seasonal affect on transmissibility of H1N1 or on contact patterns since the seasonality of human influenza in Vietnam is not predictable. Although clear peaks in influenza activity are observed, several peaks are observed each year and the timing of these peaks is not predictable and can occur throughout the year.

Health care capacity

Data on the number of public and private hospital beds, and the number of doctors and nurses by province in 2007 was obtained from the GSO. Number of ventilators by Province in 2007 was obtained from the Ministry of Health, Health Statistics YearBook 2007. The number of immediately available doses of oseltamivir in Vietnam in May 2009 was obtained from the Ministry of Health (not used in model).

Domestic pig, chicken and duck raising

Data on the number of households raising pigs, chickens and ducks by province were obtained from the GSO 2006 Rural, Agricultural and Fishery census. The denominator for estimating the proportion of households raising pigs, chickens and ducks by province (the total number of households) was obtained from the 1999 Population and Housing census. The number of households in 1999 by province was

inflated proportional to the increase in Province specific population observed between 1999 and 2007. Since two new Provinces were created in 2004 by splitting two single Provinces, the number of households in 1999 in each of the two new Provinces was estimated by dividing the number of households in each original Province in 1999 proportionately to the population in the new and old Provinces in 2007.

Contact between infectious humans and domestic pigs, chickens and ducks

To estimate the number of domestic pigs, chickens and ducks exposed to an H1N1 infected human we divided the total number of infected persons in each province by the average household size for each province to give a conservative estimate of the number of infected households. This is a conservative estimate since it assumes all human H1N1 cases are clustered by household. The estimated number of infected households was then multiplied by the proportion of households raising pigs, chickens and ducks and the average number of pigs, chickens and ducks present in households that raise these animals.

Interventions

We explored the potential impact of school closure by introducing a relative reduction in contact frequency among children in the age class 6 and 15 years. We explored the potential impact of broader social distancing measures by reducing contact frequency across all age classes.

Mathematical Model

An SEIR-model (Susceptible-Exposed-Infectious-Recovered) with a four-stage infectious period was used to model the core infection dynamics in each province. The model equations are

$$\dot{S}_{kl} = -\Lambda_{kl} \cdot \frac{S_{kl}}{N_l}$$

$$\dot{E}_{kl} = \Lambda_{kl} \cdot \frac{S_{kl}}{N_l} - eE_{kl}$$

$$\dot{I}_{kl,1} = eE_{kl} - 4\nu I_{kl,1}$$

$$\dot{I}_{kl,2} = 4\nu I_{kl,1} - 4\nu I_{kl,2}$$

$$\dot{I}_{kl,3} = 4\nu I_{kl,2} - 4\nu I_{kl,3}$$

$$\dot{I}_{kl,4} = 4\nu I_{kl,3} - 4\nu I_{kl,4}$$

where Λ_{kl} represents the force of infection on age class k in province l. Λ_{kl} is defined by

$$\Lambda_{kl} = \beta_k d_l \sum_{i=1}^{A} z_{ik}(\tau_1 I_{il,1} + \tau_2 I_{il,2} + \tau_3 I_{il,3} + \tau_4 I_{il,4})$$

where A=7 is the total number of age classes and the τ-variables allow for stage-specific infectivities. The variable S_{kl} represents the number of susceptible individuals in age class k currently in province l. E_{kl} represents exposed individuals and $I_{kl,s}$ represents infected individuals in stage s (out of a total of four) of their infection.

The parameters τ_k were all set to one since we could not find good information on shedding and infection duration at the time the model analysis was run. The parameters β_k represent age-specific susceptibility and can be found in Table S3. Parameters d_l represent province-specific population density and were computed as outlined on page 1 of this supplement. N_l is the population of province l. Parameters z_{ik} are mixing rates between age class i and age class k (from contact rate matrix on page 2). The parameter v is the recovery rate ($1/v$ is the duration of infection), and $1/e$ is the length of exposure before a host becomes infectious.

Migration and hospitalization were integrated stochastically into the above differential-equations model. Once a day, discrete individuals could move from one province to another according to the migration matrix outlined earlier; migration probabilities were balanced between provinces so the system behaved like a gravity model. In addition, once a day, discrete individuals in stage 4 of their infection could

be hospitalized based on their age-dependent probability of hospitalization and the availability of hospital beds in their province. Hospitalization time was five days. Numbers of hospitalized and migrated individuals were drawn from a Poisson distribution.

The basic reproduction ratio R_0 for the entire model can be calculated with standard next-generation matrix methods (Diekmann and Heesterbeek, 2000). Assuming at most one cross-province migration event during a single infection, a 448 by 448 matrix can be built showing the expected number of infections generated by an individual in age class k and location l in individuals of age class k' and in location l. If m is the probability of migrating from location l to any other location l' during the course of an infection of length $1/v$, then

$$L = (1 - m) \times (1/v) + m \times (1/2v)$$

is the duration of time spent infectious in location l. Then,

$$P_T \times L \times \beta_{k'} \times d_l \times z_{kk'} \times N_{kl}$$

is the number of infections generated at location l in age class k', by an infectious individual in location l of age class k. N_{kl} is the number of individuals in age class k at location l, and P_T is a proportionality constant that depends on the unknown probability of transmission given contact. A similar calculation can be done when the two locations are not equal, and a complete next-generation matrix can be filled in. This assumes only one migration event during a course of infection, but this is a fair approximation since daily migration probabilities are small and infections are short. Note that some other parts of this computation are necessarily approximate since part of the model occurs in continuous time with fractional individuals, while another part (migration, hospitalization) occurs at discrete time points and concerns whole individuals.

The R_0 value described in the figures and text is for a hypothetical Ho Chi Minh City with no cross-province emigration, and model results are presented for a single case introduced in Ho Chi Minh City. The value $R_{0,ng}$ calculated for the model via the next-generation matrix (as above) will usually be 5% to 15% lower than this $R_{0,HCMC}$ computed for Ho Chi Minh City with no migration. $R_{0,HCMC}$ is presented in the paper

since (1) we do not know the true rate of migration, (2) sick individuals may not migrate in reality, and (3) true migration probably takes place under a "residency model" (where individuals reside in one place, migrate, then return) rather than under a gravity model.

It is known that transmissibility varies from person to person and contact rates vary from community to community; thus, choosing a single R_0 value for an entire population can prove difficult. As a reference point, we use the R_0 value for a hypothetical Ho Chi Minh City with spatially uniform population density, spatially uniform interpersonal contact behavior, and no emigration. The R_0 values for the other provinces are smaller by some amount, depending on each province's population density and age structure.

Sensitivity analysis

The varied parameters with ranges were: $1.2 < R_0 < 3.1$; 3.8 days < duration of infection < 5.5 days; 1.35% < daily probability of migration < 5.00%; 0.5 < traffic on small road relative to large road < 1.0; 0.5% < probability of hospitalization < 1.5%. 1000 parameter sets were drawn randomly from this range using Latin hypercube sampling and medians and quartile ranges are presented from these 1000 runs [11].

Daily probability of migration was derived from the Ha Nam survey – which gave a mean estimate of 1.35% of people moving province each day; this was used as the lower end of the modeled range.

References

1. Del Valle SY, Hyman JM, Hethcote HW, Eubank SG: **Mixing patterns between age groups in social networks**. *Social Networks* 2007, **29**:539-554.
2. Mossong J, Hens N, Jit M, Beutels P, Auranen K, Mikolajczyk R, Massari M, Salmaso S, Tomba GS, Wallinga J *et al*: **Social contacts and mixing patterns relevant to the spread of infectious diseases**. *PLoS Med* 2008, **5**(3):e74.
3. Wallinga J, Teunis P, Kretzschmar M: **Using data on social contacts to**

estimate age-specific transmission parameters for respiratory-spread infectious agents. *Am J Epidemiol* 2006, **164**(10):936-944.

4. Fraser C, Donnelly CA, Cauchemez S, Hanage WP, Van Kerkhove MD, Hollingsworth TD, Griffin J, Baggaley RF, Jenkins HE, Lyons EJ *et al*: **Pandemic Potential of a Strain of Influenza A (H1N1) : Early Findings**. *Science* 2009.
5. Lessler J, Reich NG, Brookmeyer R, Perl TM, Nelson KE, Cummings DA: **Incubation periods of acute respiratory viral infections: a systematic review**. *Lancet Infect Dis* 2009, **9**(5):291-300.
6. Cauchemez S, Carrat F, Viboud C, Valleron AJ, Boelle PY: **A Bayesian MCMC approach to study transmission of influenza: application to household longitudinal data**. *Stat Med* 2004, **23**(22):3469-3487.
7. Boelle PY, Bernillon P, Desenclos JC: **A preliminary estimation of the reproduction ratio for new influenza A(H1N1) from the outbreak in Mexico, March-April 2009**. *Euro Surveill* 2009, **14**(19).
8. Nishiura H, Castillo-Chavez C, Safan M, Chowell G: **Transmission potential of the new influenza A(H1N1) virus and its age-specificity in Japan**. *Euro Surveill* 2009, **14**(22).
9. **Update: novel influenza A (H1N1) virus infections - worldwide, May 6, 2009**. *MMWR Morb Mortal Wkly Rep* 2009, **58**(17):453-458.
10. **Serum cross-reactive antibody response to a novel influenza A (H1N1) virus after vaccination with seasonal influenza vaccine**. *MMWR Morb Mortal Wkly Rep* 2009, **58**(19):521-524.
11. Blower SM, Dowlatabadi H: **Sensitivity and Uncertainty Analysis of Complex Models of Disease Transmission: An HIV Model, as an Example**. *International Statistical Review* 1994, **62**(2):229-243.
