# Supplementary figures and images for: Modelling the progression of pandemic influenza A (H1N1) in Vietnam and the opportunities for reassortment with other influenza viruses
Source: BMC Med. 2009 Sep 3;7:43. doi: 10.1186/1741-7015-7-43 (PMC2753341; doi:10.1186/1741-7015-7-43)

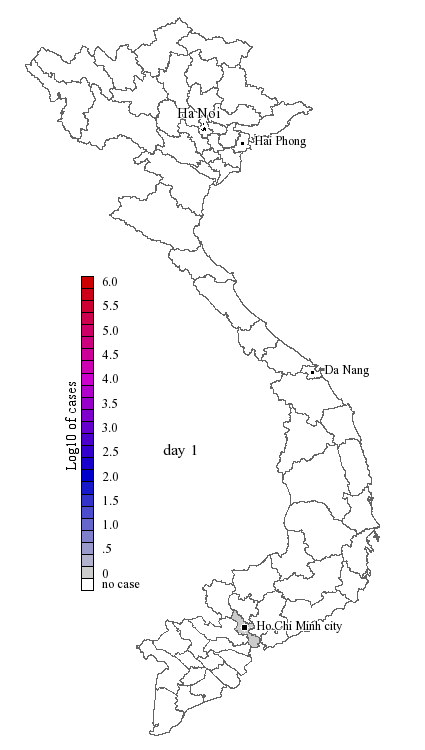

Supplement: Additional file 2 — Geographic spread of swine-origin influenza A (H1N1) in Vietnam. Animated GIF file that shows the full day-by-day epidemic shown in Figure 2. [file 1741-7015-7-43-S2.gif]

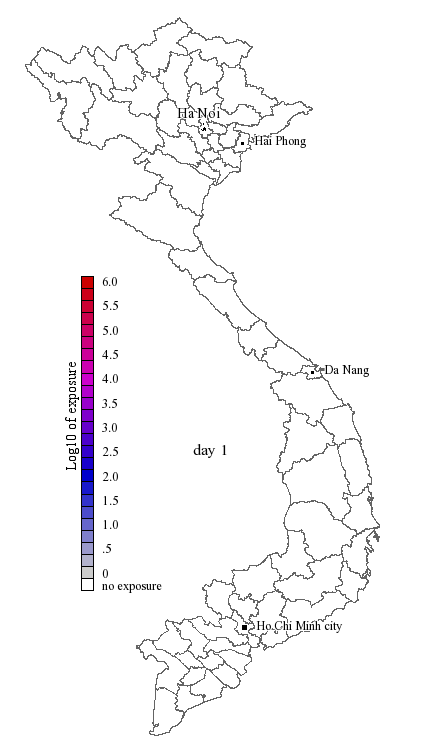

Supplement: Additional file 3 — Geographic timeline of chicken exposure during an influenza epidemic in Vietnam. Animated GIF file that shows the full day-by-day exposure of chickens to human influenza infections shown in Figure 4. [file 1741-7015-7-43-S3.gif]
